# Supplementary figures and images for: The bone phenotype associated with cherubism is independent of Caspase-1-dependent inflammasome activation in the mouse
Source: PLoS One. 2025 Feb 14;20(2):e0318826. doi: 10.1371/journal.pone.0318826 (PMC11828375; doi:10.1371/journal.pone.0318826)

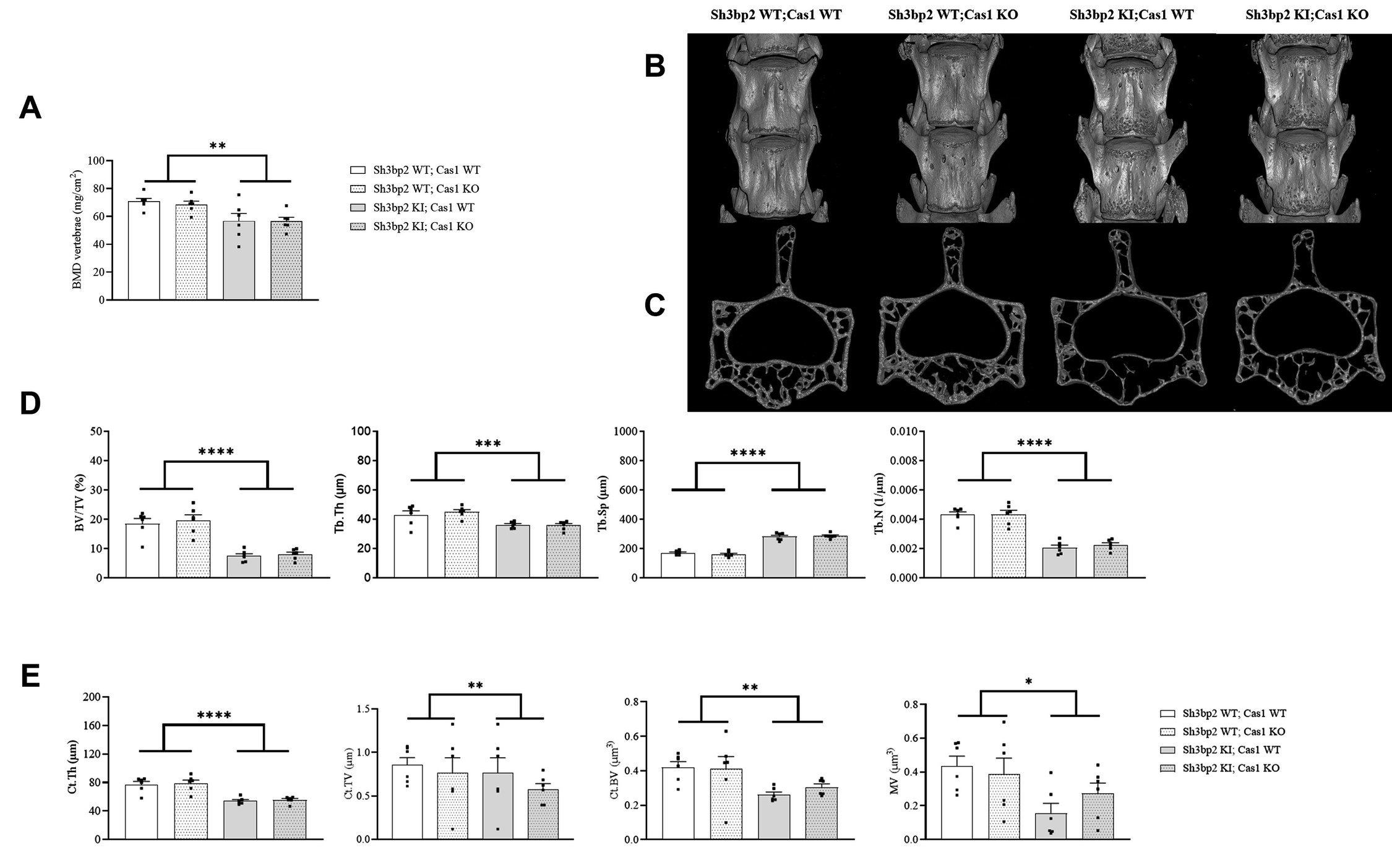

Supplement: S1 Fig — A. Male vertebral BMD for each genotype. B. Representative 3D µ CT reconstructions of vertebrae showing multiple osteolytic lesions. C. Representative 3D μCT coronal reconstructions of male mouse vertebrae at 10 weeks of age for each genotype. D. Microarchitecture analysis of trabecular parameters (BV/TV = Bone volume/Tissue volume; Tb.Th = trabecular thickness; Tb.Sp = trabecular separation; Tb.N = trabecular number) (n = 6/group). E. Microarchitecture analysis of cortical parameters (Ct.Th = cortical thickness; Ct.TV = cortical tissue volume; Ct.BV = cortical bone volume; MV = medullary volume) (n = 6/group). Values are presented as dots and mean ± SEM. Statistical analysis was performed by one two-way ANOVA. Statistical significance was set at * p < 0.05, **p < 0.01, ***p < 0.001, ****p < 0.0001. (TIF) [file pone.0318826.s001.tif]

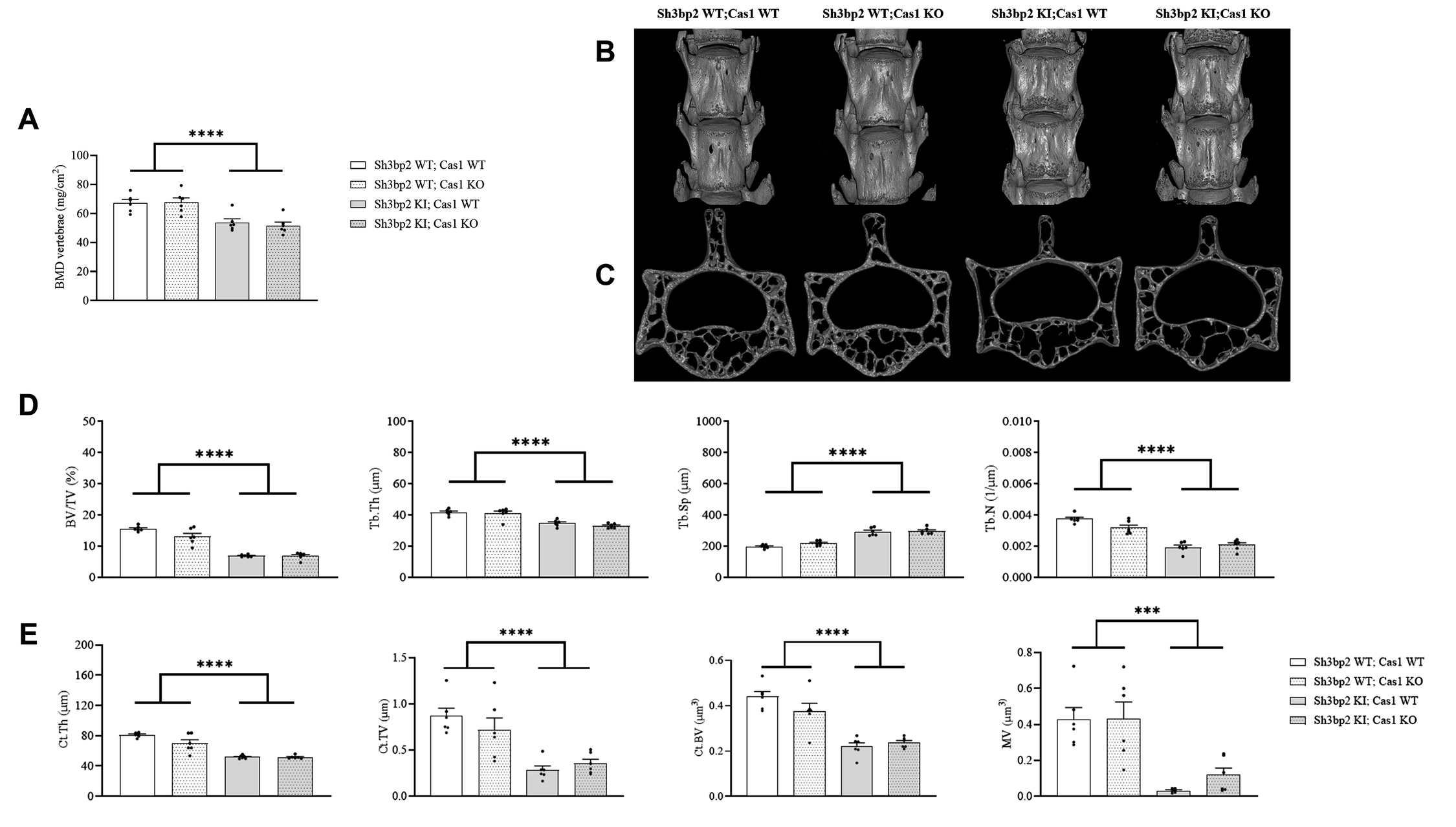

Supplement: S2 Fig — A. Female vertebral BMD for each genotype. B. Representative 3D µ CT reconstructions of vertebrae showing multiple osteolytic lesions. C. Representative 3D coronal μCT reconstructions of female mouse vertebrae at 10 weeks of age for each genotype. D. Microarchitecture analysis of trabecular parameters (BV/TV = Bone volume/Tissue volume; Tb.Th = trabecular thickness; Tb.Sp = trabecular separation; Tb.N = trabecular number) (n = 6/group). (C) Microarchitecture analysis of cortical parameters (Ct.Th = cortical thickness; Ct.TV = cortical tissue volume; Ct.BV = cortical bone volume; MV = medullary volume) (n = 6/group). Values are presented as dots and mean ± SEM. Statistical analysis was performed by two-way ANOVA. Statistical significance was set at ***p < 0.001, ****p < 0.0001. (TIF) [file pone.0318826.s002.tif]

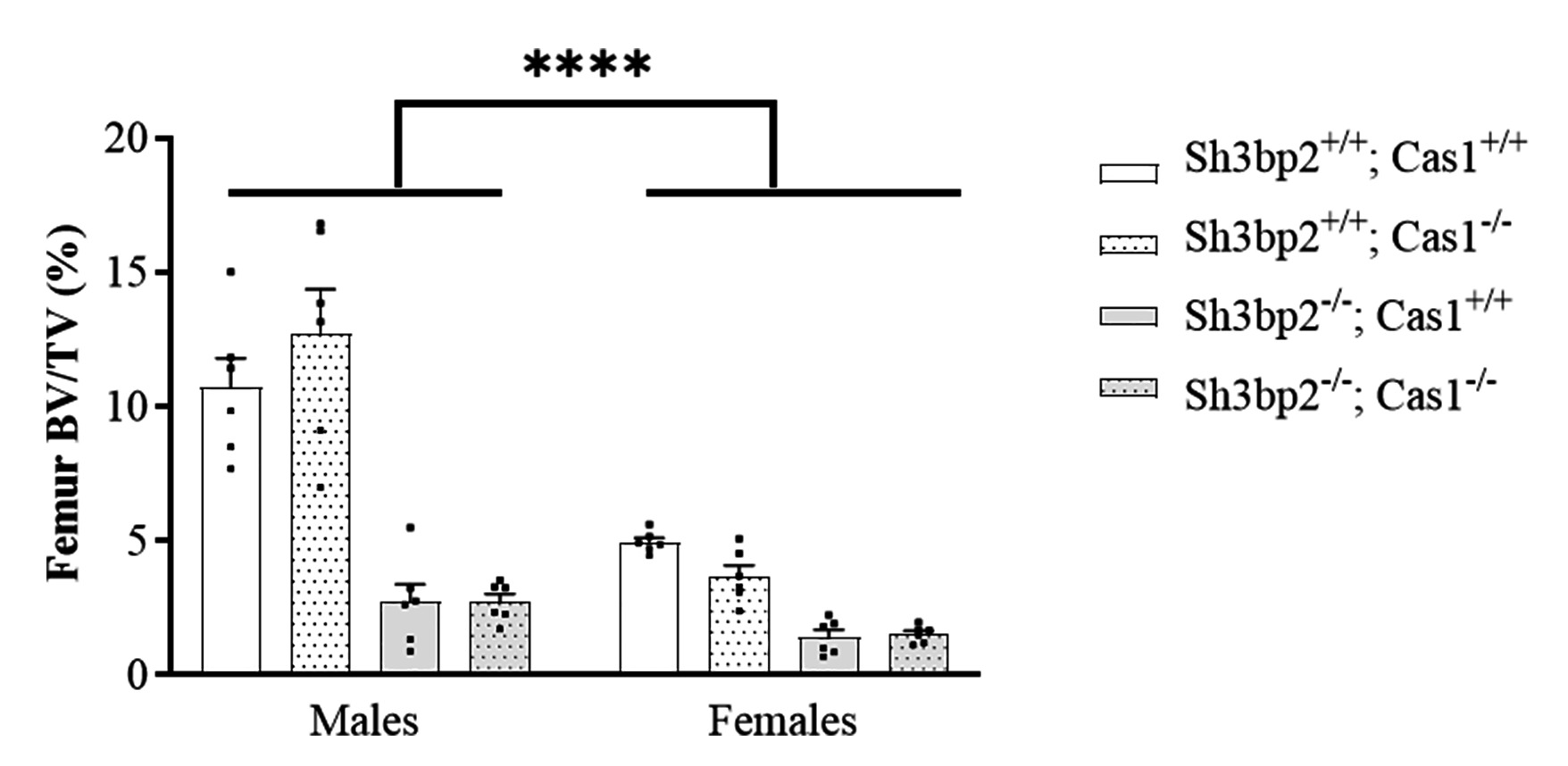

Supplement: S3 Fig — Femur BV/TV was measured in 10-week-old male and female mice for each genotype (n = 6/group). Values are presented as dots and mean ± SEM. Statistical analysis was performed using three-way ANOVA. Statistical significance was set at ****p < 0.0001 (male BV/TV vs. female BV/TV). (TIF) [file pone.0318826.s003.tif]
