# Supplementary material for: The bone phenotype associated with cherubism is independent of Caspase-1-dependent inflammasome activation in the mouse
Source: PLoS One. 2025 Feb 14;20(2):e0318826. doi: 10.1371/journal.pone.0318826 (PMC11828375; doi:10.1371/journal.pone.0318826)
Supplement: Attachment — Submitted filename: renamed_9dc04.docx [file pone.0318826.s005.docx]

We would like to express our sincere gratitude to the reviewers for their valuable comments.

You will find a point-by-point response to all the feedback we received on our paper.

The paper was edited for English by an English native spoken person.

**Reviewer #3: The authors have satisfactorily addressed most of my previous concerns. However, the following remaining concerns may require additional attention.

1. The authors performed 3-way ANOVA analyses on BV/TV and showed no significant sex-dependent difference or interaction among Sh3bp2 KI, Caspase-1 KO, and sex. This information should be included in the revised manuscript.**

We have included this information in supplemental data.

**However, because the immediate downstream effect of Caspase-1 KO would be IL-1β. Specifically, as I have pointed out in my original review, the increase in serum IL-1β level in female Sh3bp2 KI mutants was much greater than that in male Sh3bp2 KI mutants. Similarly, the Cas 1 KO-related suppression in the increase in serum IL-1β level in female Sh3bp2 KI mutant was much larger than that in male mutant mice (Fig. 3). Accordingly, two-way or three-way ANOVA should also be performed on serum IL-1β to definitively rule out a sex-dependent effect.**

We want to kindly remind the review that we had added such a graph in the previous answer. We share it here again.

The 3-way anova conducted to analyze those data gave this results :

| Source of Variation | % of total variation | P value | P value summary | Significant? |  |
| --- | --- | --- | --- | --- | --- |
| Sex | 2,191 | 0,3081 | ns | No |  |
| SH3BP2 | 4,609 | 0,1426 | ns | No |  |
| CASPASE | 8,313 | 0,0518 | ns | No |  |
| Sex x SH3BP2 | 0,7539 | 0,5479 | ns | No |  |
| Sex x CASPASE | 2,556 | 0,2715 | ns | No |  |
| SH3BP2 x CASPASE | 4,420 | 0,1508 | ns | No |  |
| Sex x SH3BP2 x CASPASE | 2,685 | 0,2599 | ns | No |  |
|  |  |  |  |  |  |
| ANOVA table | SS (Type III) | DF | MS | F (DFn, DFd) | P value |
| Sex | 685,9 | 1 | 685,9 | F (1, 34) = 1,071 | P=0,3081 |
| SH3BP2 | 1443 | 1 | 1443 | F (1, 34) = 2,253 | P=0,1426 |
| CASPASE | 2603 | 1 | 2603 | F (1, 34) = 4,063 | P=0,0518 |
| Sex x SH3BP2 | 236,0 | 1 | 236,0 | F (1, 34) = 0,3685 | P=0,5479 |
| Sex x CASPASE | 800,2 | 1 | 800,2 | F (1, 34) = 1,249 | P=0,2715 |
| SH3BP2 x CASPASE | 1384 | 1 | 1384 | F (1, 34) = 2,161 | P=0,1508 |
| Sex x SH3BP2 x CASPASE | 840,7 | 1 | 840,7 | F (1, 34) = 1,313 | P=0,2599 |
| Residual | 21778 | 34 | 640,5 |  |  |

So there is no effect of the 3 factors tested, and no significative difference between males and females

**In addition, I also disagree with the authors that bone phenotype analysis is never conducted comparing males and females as females have lower bone density or BV/TV. On the contrary, many sex-dependent differences (for example by two-way ANOVA) in various bone phenotypes were analyzed, identified, and recently reported. I also disagree with the authors that presentation of data on males and females on the same figure is not the standard way to do in the bone field.**

We think there is a misunderstanding. We have no trouble to present the males and females data in the same figure, as the reviewer can observe in the new version of the figures.

Concerning the analysis of males and females in the same graph, we agree with the reviewer, that it might have been recently reported that way. We just prefer not to, for simplification purposes.

**2. I still have issues with the authors’ decision to combine the two Sh3bp2 WT groups (with or without Cas 1 KO) as a single group and the two Sh3bp2 KI groups (with or without Cas 1 KO) as a single group for comparison in the various figures. This way of analyses assumed that the contribution of Cas 1 deficiency was not essential in the Sh3bp2 KI bone phenotype. Since the original hypothesis was to predict a significant role of Cas 1 deficiency on the bone phenotype, the combination of groups with and without Cas 1 KO is inappropriate. The authors have not provided a justification or rationale for their decision to combine the with and without Cas 1 KO as a single treatment for statistical analysis.**

We can understand the reviewer’s concerns about the way we conducted the statistical analysis of our data. However, we conducted 2-way ANOVA analysis to explore the effect of the Caspase-1 deletion and the SH3BP2 KI. We honestly don’t know how we could have done it differently. If the reviewer wants to suggest another way to analyze the data, we would be more than happy to discuss this issue further in order to satisfy the reviewer.

**3. The word Cas 1“invalidation” is vague and inappropriate. The authors still choose to use Cas 1 invalidation to describe deletion of Cas 1 throughout the revised manuscript.**

The reviewer is right. We didn’t pay enough attention. We apologize. We removed all the invalidation and replaced it by deletion.

**4. The primer listed in Table 1 needs additional important information. For example, please indicate which of them were 5’-primers and which were 3’-primers.**
The reviewer is right. We forgot to give these important information. It has been corrected.

**5. Although some people use 70% ethanol as fixation agent for bone histomorphometry, I strongly believe that it is not a good fixation method as 70% ethanol can dehydrate specimens, dissolve proteins, lipids and alter cellular and organelle as well as collagen matrix structures. Formalin would be a better fixation agent.**
We think that there is a misunderstanding regarding the use of 70% ethanol. We agree with the reviewer that 70% ethanol is not the best fixative agent. We use 70% for our histomorphometry analysis in MMA sections especially because our protocols were designed like that, but we use formalin when our samples are embedded in paraffin.

**6. The authors did not address my previous question as to whether only TRAP+ cells on bone surface counted as osteoclasts. This information is important because large numbers of TRAP+ non-osteoclastic cells were found in the marrow space.**

We thought we did in the material and methods. We modified the sentence to be clearer.

**7. The authors have indicated that a new table summarizing the Caspase 1 KO bone phenotype data was added to the Discussion. However, no such table is found in the revised manuscript or in the Discussion section.**

It was a mistake and we indeed didn’t add any table. We apologize.

**8. The resolution of all figures is still rather poor.**

The reviewer is right. We have noticed that the resolution of all figures in the generated PDF is still poor. However, this was out of our control. If you click on the figure, the quality is much improved.
