# Supplementary material for: The bone phenotype associated with cherubism is independent of Caspase-1-dependent inflammasome activation in the mouse
Source: PLoS One. 2025 Feb 14;20(2):e0318826. doi: 10.1371/journal.pone.0318826 (PMC11828375; doi:10.1371/journal.pone.0318826)
Supplement: Attachment — Submitted filename: renamed_49fa0.docx [file pone.0318826.s004.docx]

We would like to express our sincere gratitude to the reviewers for their valuable comments.

You will find a point-by-point response to all the feedback we received on our paper.

**The authors are required to address the following points during the revision.**

**1) Evaluate the phenotype of other skeletal sites besides jaw bones**

We have added the analysis of bone loss in the mandible. It was conducted as previously described by Yoshimoto *et al.* for example. We have added the analysis protocol in the materials and methods section, and add a paragraph in the results section.

**2) Perform data analysis to evaluate sex-dependent effects of Sh3bp2 KI mutation**

We conducted such analysis in order to answer the comment of the reviewer #3. However, comparing males and females does not make scientific sense as the females have by nature a lower bone density. In the bone field, males and females are always analyzed separately.

**3) Perfrom ANOVA to confirm conclusions drawn based on T-test**

In the materials and methods, we described the two-way ANOVA we conducted. The mention to a one-way ANOVA was a mistake copy and paste in all the figures.

**4) Provide legible, high resolution figures**

We modified the quality of the figures.

**5) Revise text to eliminate misinterpretation of data and conclusions not supported by experimental data**

We modified the discussion section.

**6) Careful editing of the manuscript to correct spelling and grammar.**

The manuscript was edited for spelling and grammar.

**In addition to the above required changes, the authors are also recommended to provide an indepth discussion for the lack of skeletal phenotype in the mutant mice.**

We improved the discussion in this sense.

**Reviewer #1**: **The Figures in PDF version of this manuscript are pixelated and hard to recognize, especially Figure 4, it is totally black.**

**Therefore it is impossible to judge scientific merits of the work reported in this manuscript.**

The figures were downloaded in a better quality especially in the pdf generated by PLOS One.

**Reviewer #2:** **This study focused on the involvement of IL-1 β in the cherubism pathogenesis, and the goal was to demonstrate that knocking down the expression of caspase 1 (Cas1) in Sh3bp2 knock-in (Sh3bp2 KI) mice will reverse the effect of cherubism on bone phenotype. For this purpose, they used Sh3bp2 Knock-In (Sh3bp2 KI) and Caspase1 knockout (Cas1 KO) mouse models. However, the results did not show any change in bone phenotype or any other parameters in the double mutant Sh3bp2 KI,Cas1 KO mice. Cherubism is a rare pediatric disease characterized by jaw osteolysis. Beginning in early childhood, both the lower jaw (the mandible) and the upper jaw (the maxilla) become enlarged as bone is replaced with painless, cyst-like growths. These growths give the cheeks a swollen, rounded appearance and often interfere with normal tooth development. In some people the condition is so mild that it may not be noticeable, while other cases are severe enough to cause problems with vision, breathing, speech, and swallowing.**

**- This is the first study that analyzed the phenotype of this Cherubism mutation,** **G418R KI, in mice. However, there is a major problem with this study. While TNF level in Sh3bp2-KI mice was almost-10-fold greater compared to WT mice, the level of Il-1beta in Sh3bp2-KI mice did not change much from WT mice (Fig. 1), and the authors did not explain the reason why the study focused on IL-1beta instead of TNF, and the graph in fig. 1 does not show if there is a significant difference between the level of Il-1beta in WT and Sh3bp2-KI mice.**

We did not focus on TNFα because it has extensively been done by Ueki et al in their original paper describing for the first time the Sh3bp2 KI mouse as a cherubism mouse model and the central role of TNFα role in the development of the cherubism phenotype in mouse. We wanted to explore the potential involvement of IL-1β in the mouse cherubism phenotype as some patients have increased IL-1β serum levels.

**- In Fig. 6.A. and Sup. Fig 8. Representative pictures of TRAP staining of primary osteoclast cultures from spleen from different genotypes. There are very few multinucleated Trap positive cells in WT spleen cells after 14 days treatment with MCSF and RANKL, and it is not obvious if the large cells from mutants are multinucleated or not. The authors did not explain why they chose 14 days, while it is known that splenocytes need 3-4 days to form Trap positive cells. Then, on day 4, mononuclear TRAP-positive cells will start to fuse to form multinuclear TRAP-positive cells, and after 7 days, multinuclear TRAP-positive cells may go through apoptosis.**

We use this protocol of osteoclast differentiation in our laboratory for quite some time now. There are as many osteoclast differentiation protocols as laboratory working in osteoclasts. Our protocol might not be the most efficient but is the working one in our hands.

**- The paper is poorly written and needs to be revised and the English needs improvement.**

The paper was edited for English by an English native spoken person.

**Reviewer #3**: **Cherubism, a rare dominant autosomal pediatric autoinflammatory disease characterized by a loss of bone mass with fibrous tissues replacement that was restricted to jaw bones. It is caused primarily by gain-of-function mutations on Sh3bp2 gene that led to increased secretion of TNFα. The authors found that 10 weeks-old Sh3bp2 knockin (KI) transgenic mice not only developed Cherubism but IL-1β, which plays a central role in autoinflammatory diseases. This study sought to test the hypothesis that Caspase-1-mediated IL-1β secretion is involved in Cherubism by testing whether deletion of Caspase-1 would rescue the Cherubic bone phenotype in Sh3bp2 KI mutant mice. It was found that deletion of Caspase-1 did not reverse the abnormal bone phenotype of Sh3bp2 KI mice. The investigators concluded that the Caspase-1-mediated increased secretion of IL-1β played no roles in Cherubism and that the source of the elevated serum IL-1β may have come from Caspase-1-independent non-canonical inflammasome pathways.**

**This study has several significant deficiencies as summarized in the following:**

**1. With the exception the finding of an elevated serum IL-1β level in Sh3bp2 KI mice, the findings in this manuscript were not entirely new. In addition, the findings of this study in fact disproved the investigators’ original concept that Caspase-1-mediated released IL-1β has an important regulatory role in the abnormal bone and inflammatory phenotype in Sh3bp2 KI mutant mice. Thus, this study is of negative nature. Consequently, the clinical and scientific advances brought about by this study are incremental and only marginal.**

We demonstrated for the first time that 1. the IL-1 β serum level is elevated in the Sh3bp2 KI mouse, 2. this elevated serum level is not instrumental in the cherubism phenotype in mice, 3 and not only involved the NLRP3 inflammasome as the caspase-1 invalidation only blunt and not prevent the IL1 β serum level increase, 4. the mouse cherubism bone loss is also observed in the vertebrae. We agree that our study is negative in nature but we also added some new data on the cherubism phenotype.

**2. In humans, the bone phenotype of Cherubism is largely restricted to the jaw bones, i.e., maxilla and mandible bones. Accordingly, although there was evidence in this and other studies that Sh3bp2 KI mutant mice also caused bone loss at other skeletal sites, it is disappointing that the investigators elected not to examine maxillomandibular bones and focused on other bone sites. Investigation of maxillomandibular bone could disclose additional key information as to whether the bone phenotype in these mutant mice was similar to that seen in humans. If the effects on maxillomandibular bones were not greater than other bone sites, it follows that expression of gain-of function mutant of Sh3bp2 in mice might be different from that in humans. If so, it would suggest that this mutant mouse model might not be a suitable animal model for investigation of the pathophysiology of Cherubism in humans. As a result, the lack information on maxillomandibular bones represents a significant weakness of this manuscript.**

We have added the data concerning the mandibular bone loss. As all model, this cherubism mouse model is not perfect but is useful and used for several years.

**3. An important deficiency of this manuscript is the lack of attention to the potential sex-dependent effects by not directly comparing the effects between male and female mice. Accordingly, it is important to document whether there were sex-dependent differences. [Some of the findings (see comment 5 below) might have shown significant sex-dependent differences]. In this regard, the data on male mice and those on female mice should be combined as a single Figure. This would then not only allow direct comparison between male and female mice but would also allow the determination whether there is a sex- and genotype-dependent interaction with two-ways ANOVA.**

**Therefore, Fig. 2 and Suppl. Figs. 1 and 2 should be combined as a single figure. [Fig. 2A and Suppl. Fig. 1A were redundant, so Fig. 2A can be removed]. The same is true for Fig. 3 and Suppl. Fig. 3, Fig. 4 and Suppl. 4, Fig. 5 sand Suppl. Fig. 5, as well as Fig. 6 and Suppl. Fig. 7.**

**Consequently, this deficiency renders the analysis of the data incomplete.**

Bone phenotype analysis is by essence never conducted comparing males and females as females have always lower bone density or BV/TV for instance. However, we did analyze the effect of the genotype and sex for the BV/TV as the reviewer can appreciate on the following figures.

The 3-way anova conducted to analyze those data gave this results :

| Source of Variation | % of total variation | P value | P value summary | Significant? |  |
| --- | --- | --- | --- | --- | --- |
| Sex | 2,191 | 0,3081 | ns | No |  |
| SH3BP2 | 4,609 | 0,1426 | ns | No |  |
| CASPASE | 8,313 | 0,0518 | ns | No |  |
| Sex x SH3BP2 | 0,7539 | 0,5479 | ns | No |  |
| Sex x CASPASE | 2,556 | 0,2715 | ns | No |  |
| SH3BP2 x CASPASE | 4,420 | 0,1508 | ns | No |  |
| Sex x SH3BP2 x CASPASE | 2,685 | 0,2599 | ns | No |  |
|  |  |  |  |  |  |
| ANOVA table | SS (Type III) | DF | MS | F (DFn, DFd) | P value |
| Sex | 685,9 | 1 | 685,9 | F (1, 34) = 1,071 | P=0,3081 |
| SH3BP2 | 1443 | 1 | 1443 | F (1, 34) = 2,253 | P=0,1426 |
| CASPASE | 2603 | 1 | 2603 | F (1, 34) = 4,063 | P=0,0518 |
| Sex x SH3BP2 | 236,0 | 1 | 236,0 | F (1, 34) = 0,3685 | P=0,5479 |
| Sex x CASPASE | 800,2 | 1 | 800,2 | F (1, 34) = 1,249 | P=0,2715 |
| SH3BP2 x CASPASE | 1384 | 1 | 1384 | F (1, 34) = 2,161 | P=0,1508 |
| Sex x SH3BP2 x CASPASE | 840,7 | 1 | 840,7 | F (1, 34) = 1,313 | P=0,2599 |
| Residual | 21778 | 34 | 640,5 |  |  |

So there is no effect of the 3 factors tested, and no significative difference between males and females

We conducted the same 3 – way ANOVA on the femur BV/TV as the reviewer can appreciate here :

As expected, there is a significative effect of the sex and of Sh3bp2, but not of Caspase 1 KO, as the data are the following :

| Source of Variation | % of total variation | P value | P value summary | Significant? |  |
| --- | --- | --- | --- | --- | --- |
| Sex | 24,66 | <0,0001 | **** | Yes |  |
| SH3BP2 | 46,01 | <0,0001 | **** | Yes |  |
| Caspase1 | 0,06125 | 0,6890 | ns | No |  |
| Sex x SH3BP2 | 12,38 | <0,0001 | **** | Yes |  |
| Sex x Caspase1 | 0,8436 | 0,1424 | ns | No |  |
| SH3BP2 x Caspase1 | 0,03295 | 0,7690 | ns | No |  |
| Sex x SH3BP2 x Caspase1 | 0,9423 | 0,1217 | ns | No |  |
|  |  |  |  |  |  |
| ANOVA table | SS | DF | MS | F (DFn, DFd) | P value |
| Sex | 225,8 | 1 | 225,8 | F (1, 40) = 65,45 | P<0,0001 |
| SH3BP2 | 421,3 | 1 | 421,3 | F (1, 40) = 122,1 | P<0,0001 |
| Caspase1 | 0,5609 | 1 | 0,5609 | F (1, 40) = 0,1626 | P=0,6890 |
| Sex x SH3BP2 | 113,4 | 1 | 113,4 | F (1, 40) = 32,87 | P<0,0001 |
| Sex x Caspase1 | 7,725 | 1 | 7,725 | F (1, 40) = 2,239 | P=0,1424 |
| SH3BP2 x Caspase1 | 0,3018 | 1 | 0,3018 | F (1, 40) = 0,08746 | P=0,7690 |
| Sex x SH3BP2 x Caspase1 | 8,629 | 1 | 8,629 | F (1, 40) = 2,501 | P=0,1217 |
| Residual | 138,0 | 40 | 3,450 |  |  |

However, we modified the data presentation according to the reviewer’s comment and we presented in parallel the data obtained for the males and the females in the figures.

Obviously, if the reviewer whishes we always can present the male and the female mice in the same graph, however, it is not the standard way to do it in the bone field.

**4. In all figures, the statistical analyses among the treatment groups combined the two Sh3bp2 WT groups (with or without Cas 1 KO) and the two Sh3bp2 KI groups (with or without Cas 1 KO) for comparison by t-test. This type of analyses completely ignored the contribution of Cas 1 deficiency and would have to assume that Cas 1 KO was not essential in the Sh3bp2 KI bone phenotype. This assumption completely contradicts the original hypothesis. Consequently, the statistical analysis of this study is inappropriate. All four groups should be separately compared and analyzed with ANOVA.**

As stated in the Materials and Methods section, the analysis of the 4 groups were conducted by a two-way ANOVA. The mention of the one-way ANOVA in the figure legends was an inappropriate copy/paste. We apologize for this mistake.

**5. Many of the findings appeared to mis-interpreted, which led to erroneous conclusions in the manuscript. For** **example, there is no evidence in this manuscript supporting the conclusion that “these data might suggest that the increased production of IL-1β is involving the non-canonical inflammasome pathway and other caspases for this production”. On the contrary, the serum level of Sh3bp2 KI; Cas 1 KO double mutant mice was similar to that in Sh3bp2 KI WT mice (Fig. 3B and Suppl Fig. 3B). This piece of data would suggest that almost all the increased serum levels of IL-1β in both male and female Sh3bp2 KI mutant mice. Accordingly, there is no evidence for the increased serum IL-1β level in Sh3bp2 KI mutant mice was caused by any non-canonical inflammasome pathways other than the Caspase-1-dependent pathway. In addition, the increase in serum IL-1β level in female Sh3bp2 KI mutant mice was much greater than that in male Sh3bp2 KI mice. Similarly, the Cas 1 KO-related suppression in the increase in serum IL-1β level in female Sh3bp2 KI mutant was much larger than that in male mutant mice (Fig. 3B and Suppl. Fig. 3B). This would not support the conclusion of the investigators that there were no sex-dependent differences. Rather these findings suggest that there could be sex-dependent differences in these mutant mice. Finally, there was no direct measurement of osteoclastic resorption activities in this manuscript. Thus, no conclusion about bone resorption should be made.**

The reviewer is right, we have removed all statement about bone resorption as only osteoclast differentiation was analyzed.

**6. The Discussion lacks in-depth discussion or potential explanation for the lack of effects of Caspase-1-mediated IL-1β activation in the bone phenotype of Cherubism in mice.**

We have modified the discussion in accordance with the reviewer’s comment.

**7. There were many typos, syntax errors and structural mistakes, as well as misuses of several English words throughout the manuscript (Please see specific comments for examples). The manuscript would benefit from careful editing for English by an English-speaking individual.**

The manuscript has been edited for English by an English-speaking person.

**Specific comments:**

**1. Lines 1-2. The last word of the title should either be “mice” or “the mouse”. Also, it should be revised to indicate that it was independent of “the Caspase-1-dependent” inflammasome activation to be more specific because there are many types of inflammasomes.**

The sentence was modified according to the comment of the reviewer.

**2. Line 27. The word “invalidated” is vague and an inappropriate word. A better word would be “deleted” or “inactivated”. The same is true for many places throughout the manuscript.**

We removed ‘invalidated’ from the manuscript.

**3. Lines 30-32. The conclusion that “The source of the elevated IL-1β need more analysis but might come from the inflammasome noncanonical pathway, involving other caspases” was not tested. Thus, it should be a key conclusion and thereby should not be included in the Abstract.**

We remove this sentence from the Abstract.

**4. Line 58. The word “scares” should be “scarce”.**

We corrected it.

**5. Line 61. The word “funding” should be “founding”.**

We corrected it.

**6. Line 68. The word “implies” should be “involves”.**

We corrected it.

**7. Line 73. The sentence is not correct, because the study investigated the effects of deletion of Caspase-1 and not directly on IL-1β.**

We corrected the sentence.

**8. Lines 82-86. The breeding scheme for double mutants was not clear and should provide additional details. Genotyping methods for each mutant strain also need to be provided.**

We added the genotyping methods and a table for the list of primers that was used. We added details for the breeding scheme. It was a simple scheme, breeding double heterozygous animals (Sh3bp2 Het,Cas1Het) in order to obtain the 4 groups : Sh3bp2 WT;Cas1WT, Sh3bp2WT;Cas1 KO, Sh3bp2 KI;Cas1WT, Sh3bp2KI, Cas1KO.

**9. Line 91. Were the mice anesthetized or euthanized with ketamine and xylazine? In other words, were tissue samples and blood collected in anesthetized or euthanized animals? These procedures should be performed only in euthanized animals.**

We corrected the sentence.

**10. Line 97-98. 70% ethanol is not a good fixation agent for this study, since it can dehydrate specimens, dissolve proteins, lipids and alter collagen matrix structures. Therefore, it could confound the experimental results and interpretations.**

The protocol that has been used in our laboratory for decades to perform histomorphometry on undecalcified bone specimens involves fixation of bone specimens in 70% ethanol. This approach is widely recognized and used in the scientific literature for bone histomorphometric analysis.

**11. Line 111. The word “exposition” is not an appropriate word. The more appropriate word may be “exposed for”.**

We corrected it.

**12. Line 113. It should be “was used”.**

We corrected it.

**13. Line 117. Please provide a rationale for why calvaria sutures were selected as the VOI for the μ-CT analyses.**

The bone phenotype observed in the Sh3bp2 KI cherubism mouse model has been well documented since the seminal study by Ueki et al. in 2007 in Cell Journal and is characterized by mandibular bone loss, systemic bone reduction (described particularly in the femur phenotype) and the calvarial erosion. Subsequent studies have consistently included analysis of calvarial erosion in this model (see 8,11,18,23–27 in the paper reference list). Accordingly, we have followed this standard characterization in our study. An appropriate paragraph has been added to the Discussion to explain this aspect of our analysis.

**14. Lines 127-131. At what site(s) were bone histomorphometry performed? Also, were all TRAP+ cells or only those on bone surface counted? This information is important because a large number of TRAP+ non osteoclastic cells were found in the marrow space. In addition, what does it mean “as usually done”? It is vague.**

We have incorporated the clarification and revised the sentence as suggested by the reviewer.

**15. Line 134. It should be “for” 24 hr rather than “during” 24 hr.**

We have made the necessary corrections.

**16. Lines 137-141. Please explain what was “machine learning”, and how inflammation lesions were identified. Were clusters indices of inflammation? Also, it should be “The process was as the following”.**

The inflammatory lesions were previously identified as containing macrophages (Ueki et al, 2007). Since then, the inflammation lesions pathognomonic of the mouse cherubism were analyzed as we did. First, we trained the Ilastik software, on selected sections, stained with H&E, to classify the inflammatory clusters, the normal tissue and the background. The entire new batch of sections was classified using the trained Ilastik software. This modification was added in the manuscript.

17. Line 152. All the four test groups were mutant mice. Accordingly, it is important to also include C57BL/6 wild-type mice as a control group to rule out any potential effects due to gene mutants in the Ch3bp2 KI WT and/or the Cas1 WT mice.

We used the mice with the SH3BP2WT,Caspase1WT genotype as controls. These mice are genetically comparable to the standard C57BL/6 strain.

**18. Line 156. Did you mean “the cell number in cell suspension was counted”?**

We corrected the sentence.

**19. Line 176. It states that two-way analysis of variance was performed. However, results of two-way ANOVA were not presented in this manuscript.**

As stated in the Materials and Methods section that we performed two-way ANOVA which is consistent with our analysis The references to a one-way ANOVA in all figure legends were incorrect and resulted from a copy and paste error.

**20. Line 182. It states that “increase of TNFα serum level in the Sh3bp2 KI mice is associated to an increase of the IL-1β serum level”. However, Figure 1 did not show an association between the two cytokines. In fact, there is no evidence that the levels of these two cytokines in the KI mice were associated to each other. Accordingly, the conclusion of an association is incorrect. They could be independent effects.**

The reviewer is right. We have rephrased the sentence accordingly.

**21. Lines 183-187. The canonical Caspase-1-mediated inflammasome activation pathway would also cause an increase in secretion of IL-18. Therefore, to confirm an involvement of the Caspase-1 mediated canonical inflammasome activation, the investigators may also wish to measure the serum level of IL-18.**

The reviewer is correct and actually we did measure the serum levels of IL-18. However, the levels were not detectable in our samples. Most of the results obtained were not extrapolated as they should according to the supplier. The raw data are available upon request.

**22. Lines 189, 210, and 224. “Caspase 1 deficiency” is not appropriate than “Caspase 1 absence”.**

We corrected it in the whole manuscript.

**23. Lines 194-195. What do the investigators mean when they stated that “the absence of the Caspase 1 in the Sh3bp2 KI mice did not modify the lower weight and height observed in the Sh3bp2 KI male mice or the smaller size observed in the Sh3bp2 KI male mice”? This sentence is unclear. Also, Fig. 2B did not measure height?**

The reviewer is correct. We measured the length of the mice, not the height. We have made the necessary corrections.

**24. Line 204. It should be “the serum level of TNFα was still elevated …”.**

We corrected it.

**25. Line 208. It should be “decrease”.**

We corrected it.

**26. Lines 212. A rationale for focusing systemic bone loss phenotype and not evaluating the jaw bones, i.e., maxilla and mandible bones, (which is the major bone phenotype in humans) should be provided.**

We have included an analysis of the mandibular bone loss for both males and females (Figure 4).

**27. Lines 218-219. Please provide a rationale for why studies on vertebrae were done only in male mice and not female mice.**

The studies on vertebrae were performed on both males (Supplementary Figure 7) and females (Supplementary Figure 8). All the analyses in this study were performed on both males and females.

**28. Lines 224-232. The bone resorption activity is the most important osteoclastic parameter. Increased TRAP+ cell number does not necessarily indicate an increased osteoclastic resorption activity. Therefore, why in vitro bone resorption activity (e.g., resorption pit formation assay) was not performed in Fig. 6.**

The reviewer is correct. We only wanted to demonstrated the increased osteoclast differentiation and number as it was previously described in the Sh3bp2 KI mice.

**29. Lines 234-238. This section is not related to the results or any figures or tables. Therefore, it does not belong to the Results section. It may belong to the Discussion section.**

We have removed this from the results section and included a detailed paragraph in the discussion section. In addition, we have added a table summarizing the Caspase 1 KO bone phenotype data.

**30. Lines 270-271. As indicated above, there is no evidence for the increased serum IL-1β level in Sh3bp2 KI mutant mice was caused by any non-canonical inflammasome pathways other than the Caspase-1-dependent pathway.**

We appreciate the reviewer’s comment. We only suggested this is as a possible explanation for the increased the IL-1β levels. We have revised the sentence accordingly.

**31. Lines 274-275. This reviewer does not understand the conclusion about “the relatively high basal level of IL-1β could be the sign of chronic inflammation in relation to our animal facility conditions”. Please elaborate.**

The mice were not housed in a SPF animal facility. In addition, the sanitary status revealed the presence of pineworms that could alter the inflammatory status of the mice.

**32. Line 276. This reviewer also fails to understand the conclusion about that “this study allowed us to develop a robust and powerful tool to evaluate the calvarial bone resorption”.**

We developed a new protocol for semi automatically evaluate the calvarial bone resorption and we provide all the details allowing anyone to use it.

**33. Line 291. As described in above, the conclusion that the increased IL-1β serum level is not explained by the action of Caspase-1 in the cherubism mouse model” is incorrect.**

We don’t agree with the reviewer. We invalidated Caspase-1 in the Sh3bp2 KI mice. This invalidation did not prevent the production of IL-1 β suggesting that not all of the IL-1β detected is produced by caspase-1 activity.

**34. Legend of Fig. 1. The “10 weeks” was stated three times. It is redundant. One time is sufficient. Also, the symbols **, ***, and **** are not shown in the figure. These symbols should be removed from the legend as the inclusion of which adds confusion.**

We corrected it.

**35. Fig. 2B. Symbols should be added to indicate that Sh3bp2 KI significantly reduced the body weight and the body length. In addition, it appears that Cas 1 KO reverse the Sh3bp2 KI-dependent reduction in body weight. Similarly, the symbols * and ** should be removed from the figure legend to avoid confusion.**

We corrected it in this figure and in all the following figures.

**36. Legend of Fig. 3. “Dosages” should be “Concentrations or Levels”. Also, please remove * and ** from the legend as they were shown in the figure. To be consistent, the P=0.0864 may be stated in the legend instead on the figure.**

We corrected it.

**37. The sub-labeling of legend of Fig. 4 is not consistent with what is shown in the figure. For example: B is not representative femur 3D reconstruction. C is not a representative femur 3D coronal μ-CT reconstruction; etc. Also, the symbols of * and *** should be removed from the legend as they were not shown in the figure.**

The legend of this figure was corrected.

**38. Legend of Fig. 5. Please remove symbol description *, **, and *** be removed from the figure legend.**

The legend of this figure was revised accordingly

**39. Fig. 6. It would be helpful for the reading to include arrows to indicate the osteoclasts on bone surface on panel C. Also, please remove symbols *, **, and *** from the legend as they were not shown on the figure.**

We added arrows and corrected the legend according to the reviewer’s comments.
